# The Need for Standardization, Reliability and Validity in Fundamental Roots for a Successful Problem-Based Learning Program

**DOI:** 10.15694/mep.2019.000093.1

**Published:** 2019-04-22

**Authors:** Lee HangFu, Samal Nauhria

**Affiliations:** 1 Windsor University School of Medicine

**Keywords:** Problem-Based Learning, Progress Testing, Self-assessment, Standard Setting, Best Evidence Medical Education, Outcome Assessment, Reliability and Validity

## Abstract

This article was migrated. The article was marked as recommended.

McMaster University has introduced the Problem-Based Learning (PBL) in medical education over half a century ago. Since then, hundreds of reviews and study reports have identified many critical issues affecting the success of the PBL unit. Nonetheless, we are still debating the efficacy and success of the PBL program. Over half of all medical schools globally have introduced various versions of PBL pedagogy in their medical education program achieving assorted modifications of outcomes.

In this paper, I have reviewed from many scholars and through their publications; I have identified eight important Fundamental Roots for a successful PBL program. The success of any PBL program must be evaluated as a whole from the perspective of Standardization, Reliability and Validity working synchronously between these fundamental roots and not the individual PBL unit.

The importance of Standardization must consider all the critical issues identified in many reviews and study reports. These issues are one of many factors when incorporated into the fundamental framework of a PBL program will regulate all PBL units in a unified outcome. The educational objective principles will guide the reliability of a PBL program by meeting the institutional mission and students’ career success goals. The “Assessment as Learning” should incorporate the “Holistic and Divergent Approach” and the longitudinal “Progress Testing”. These are the principle methods of evaluation to achieve a reliable and valid outcome assessment in a successful PBL program.

## Introduction

Medical education has been historically taught in a traditional classroom environment using lecture-based learning pedagogy. In the 1950s, Case Western University was the first to conceptual the Problem-Based Learning (PBL) in higher education. McMaster University was the first to introduce the PBL pedagogy to medical education in 1969. This integrative medical education has transformed the advancement of the student-based medical school curriculum. There was no raison d’être, evidence-based, or practical cognitive theoretical methodology as reported by the founders of the McMaster Medical School ( Neufeld and Barrows, 1974). In an Inventory Count, Whyte (2016) from McMaster showed that PBL represented less than 2% of all instructional methods overshadowed by 55% of the lecture-based activities. A further examination of the database showed 76 of 131 medical schools have attempted at least one PBL activity, which prompted him to suggest, “This was an innovative and disruptive pedagogy which has impacted most medical schools around the globe”.

Over the last half-century, many authors have published a plethora of reviews and published articles on the PBL curriculum outcome. These publications examined the size of the group, unit dynamics, tutor experience, and PBL assessment methods with various results ( Albanese and Mitchell, 1993; Vernon and Blake, 1993; Berkson, 1993; Nandi et al, 2000; Smits et al, 2002; Newman, 2003; Dolmans and Wolfhagen, 2005; Koh et al, 2008; Hartling et al, 2010). The medical education journals have produced literature supporting and against the effectiveness of PBL pedagogy. Their reports showed no significant difference in student knowledge attainment from those medical schools adopted PBL approaches ( Farquhar et al, 1986; Albanese and Mitchell, 1993; Vernon and Blake, 1993; Berkson, 1993; Nandi et al, 2000; Enarson and Cariaga-Lo, 2001; Hartling et al, 2010; Dolmans and Wolfhagen, 2005; Prihatiningsih and Qomariyah, 2016). Most reviews have concentrated on knowledge acquisition ( Nandi et al, 2000; Smits et al, 2002) and some compared the USMLE part 1 and 2 between the traditional Lecture-Based Learning (LBL) to the PBL (Nandi et al, Smits et al, 2002; 2000; Farquhar et al, 1986; Enarson and Cariaga-Lo, 2001). The graduate outcome competency from a PBL environment is undoubtedly a relevant goal to consider ( Koh et al, 2008). An important difference that has been observed and reported between PBL and traditional pedagogy lies in the learning environment ( Norman and Schmidt, 2000).

PBL uses trigger cases or problems in small group tutorials to generate student-centred, self-directed, and team-based active learning guided by a facilitator in a structured brain-storming environment followed by a significant amount of time for independent studies ( Lee and Kwan, 1997; Wood, 2003). Students learn under the tutor or facilitator guidance by emphasizing on existing knowledge and new information attainment from institutionally provided or student-identified learning resources ( Groves, 2005; Walsh, 2005; Leary et al, 2009).

The Seven Steps/Jumps Method is the most commonly cited PBL model described by Schmidt (1983) in Maastricht. Barrett and Cashman (2010) offered a contemporary adaptation of this model where they added a pre-step - ‘Setting the Climate’. They have also included a comprehensive summary on roles of the tutor/facilitator and students within the PBL team. Students all work on the problem while roles of chairperson, scribe, reader and timekeeper were assigned to students at the initial formation of the small group. All active PBL learning is beneficial to students in the attainment of content knowledge. The student will also develop an assortment of higher learning skills, outcome competencies, and self-awareness that is major attributes for an effective graduate medical professional ( Koh et al, 2008; Prihatiningsih and Qomariyah, 2016).

The evidence that Colliver (2000) has presented is undeniable. The author concluded that PBL had not improved medical student’s knowledge base and clinical performance as anticipated in the fundamental design of a PBL curriculum. Any study that treats PBL as the promise for the attainment of basic medical knowledge and clinical competency will arrive at a conclusion of minimal difference. Norman and Schmidt (2000) analyzed and concluded in the review, which differs from Colliver (2000) in two conceptual ways: First, PBL provides a challenging, encouraging and pleasurable environment to medical education. Second, the PBL will progressively advance when the program has achieved a practical and reliable pedagogy from theory building to realistic evaluation with an intentional attempt to focus on the central component of self and team education in solving a problem.

There are truths to both of these diverging theories. If we accept the Colliver’s conclusion, the PBL methodology for the last 50 years is all but a “waste of time”. On the other side of the argument, should we continue the same methodology for another 50 years, for the sake of a better learning environment without a significant advantage in student’s knowledge or Clinical Preparation? Over the last half century, there are overwhelming publications that have examined the achievements of PBL results. These reviews and meta-analysis have studied the assessments of content and objective outcomes, effects of tutor experience, learning methodologies, and the PBL unit logistics ( Farquhar et al, 1986; Albanese and Mitchell, 1993; Berkson, 1993; Lee and Kwan, 1997; Colliver, 2000; Nandi et al, 2000; Norman and Schmidt, 2000; Enarson and Cariaga-Lo, 2001; Smits et al, 2002; Wood, 2003; Newman, 2003; Groves, 2005; Dolmans and Wolfhagen, 2005; Walsh, 2005; Koh et al, 2008; Leary et al, 2009; Hartling et al, 2010; Prihatiningsih and Qomariyah, 2016). These institutional publications have concluded their findings using various educational visions, learning diversities, content acquisition, medical licensing assessments and individualized hypothetical analysis ( Azer, 2001; Tsou et al, 2009). However, none of them has assessed the PBL program from the perspective of Standardization, Reliability and Validity as required basic accreditation criteria for the purpose of medical education.

I believe PBL is a noteworthy practical learning methodology. We must reevaluate the efficacy by examining the purpose of PBL in the realm of Standardization, Reliability and Validity of its fundamental roots. In this paper, I have identified “Eight PBL Fundamental Roots”. I will review pertinent positive and negative, direct and indirect research evidence as we re-examine and re-focus the effect of Standardization, Reliability and Validity that will apply specifically to the success of any PBL program. The need to implement Standardization, Reliability and Validity must identify and challenge the aim for goal-attainment relating to the roots of the PBL program ( Albanese and Mitchell, 1993; Nandi et al, 2000; Azer, 2001; Singaram et al, 2008; Colliver, 2000).

## Basic Fundamental Roots of the PBL program

### Basic Fundamental Roots of the PBL program:

Eight Fundamental roots of any PBL program have been identified in this paper as follows:

1. The Institutional acceptance to change and adjustment to PBL ownership.
2. The PBL objectives as it reflects on institutional mission and vision.
3. Student abilities and diversities.
4. PBL learning objectives must include content Learning objectives from required disciplines.
5. Structures, Characteristics and Functions of the PBL unit.
6. The Tutor/Facilitator Experience and Competencies.
7. The objective and frequency for an effective student assessment.
8. Self PBL program assessments.

1. **The Institutional Acceptance to Change and Adjustment to PBL Ownership:**

A quote from Biggs (2007) “The reasons why PBL is not used more widely are not educational but organizational”. The PBL innovations were thought to be a mark of progress since the start of PBL educational revolution in the 1960s. In the 1970s, the PBL successes, the benefits of innovations, the follow-through and validity of its assessments were all being questioned. Fullan (1992) identified the institution as the origin to change. Leaders are involved in developmental efforts and contribute to significant organizational transformation.This will require interacting continuously between faculty, staff, and professional members to learn, work, plan, identify new-ideas, resolve problems, and assess effectiveness. The Institutional teaching culture must be changed since much of the traditional medical education is embedded in the conventional structures and routines that have been individually internalized. The nature of human tendency is to remain status-quo, achieving acceptance and change will require intensive faculty development continuously for many years. Substantial progress requires a change in an institutional organization, designating new roles, and empowering people to change before they will accept the PBL ownership within an institution.

2. **The PBL Objectives as it Reflects on Institutional Mission and Vision:**

The mission statement is the necessary myriad of activities to achieve a centrally shared purpose that expresses a sense of its educational vision guiding its students to learn and how learning can be used to benefit the society ( Meacham and Gaff, 2006). The objectives of a PBL system should focus on the development of ( Barrows, 1994; Husain, 2011):

a. Clinical reasoning skills.
b. Self-directed study skills.
c. Depth and focus of knowledge acquired.
d. Conceptual skills.
e. Team skills

Under the meaningful supervision of the facilitator, students should work as a team to generate several hypotheses to identify the Learning Objectives of the case problem. These hypotheses must affect the Basic Science Discipline content learning objectives ( Prihatiningsih and Qomariyah, 2016).Students should independently seek out the information necessary to determine, analyze, and synthesize the basic mechanisms responsible for all symptoms, signs and laboratory findings that will effectively explain the scenario involved in the PBL case ( Schmidt, 1983). The Content Learning Objectives must emphasize the “System” involved down to the molecular level and pathophysiological causes as appropriate to guide the investigation, pharmacological and surgical management into the patient’s differential diagnosis and outcome ( Barrett and Cashman, 2010).

3. **Student Abilities and Diversities:**

It is paramount and important that students and faculty are aware of the potential diverse settings in the PBL unit. Diversities are inclusive of but not limited to multicultural, multilingual, different socio-economic and educational backgrounds. Duek (2000) noted that diverse factors such as gender, religion, language, prior educational experience, and age of a student are important diversities that will affect the PBL unit effectiveness. Intercultural learning complexity can influence group participation, team cohesiveness and cognitive attainment within a small PBL discussion group ( Carlo et al, 2003).Cultural diversity and second language challenge of a student can lead to avoidance of active participation creating a dysfunctional PBL group ( Hendry et al, 2003; Gill et al, 2004). Therefore, the cultural diversity on the effectiveness of a PBL group cannot be disregarded ( McLean et al, 2006). PBL unit facilitator and members should address and be aware of these issues early to heighten group effectiveness, encourage students to overcome social and cultural barriers and prevent intra-group detriment from a diverse cultural PBL group ( Singaram et al, 2008).

4. **PBL Learning Objectives must include Content Learning Objectives from Required Disciplines:**

Barrows ( 1986, 1988, 1994) summarized six key educational PBL objectives (1) Better self-directed knowledge attainment for clinically application; (2) Assert an effective problem solving clinical skills; (3) Apply and integrate self-directed learning skills; (4) sympathize patient’s medical and psycho-social needs; (5) Derive a self-motivated and student-centred learning method, and (6) Promote independent critical evaluation skills.

When these Learning Objectives are incorporated into the PBL educational program, students seem to have achieved higher cognitive skills by utilizing the hypothesis-driven reasoning strategies while providing coherent explanations to problems including scientific concepts in their reasoning ( Groen et al, 1993; Hmelo et al, 1997). PBL students are more socially proficient in small-group cooperation, inter-collegial communication, and consultative skills ( Azer, 2001; Woods, 1994). PBL students developed critical appraisal skills with a mature understanding of self-directed learning strategy ( Kaufman and Mann, 1997).They are more likely to demonstrate the essential skills of a medical professional possesses leadership, functioning as a respectable team member, participation in peer learning, reflective attitudes, critical evaluation, identify and prioritize tasks, highly motivated, and demonstrate effective time management ( Azer, 2001).

Although the benefits are undeniable, most studies showed a mixture of graduation outcomes related to PBL curricular pedagogy ( Albanese and Mitchell, 1993; Schmidt, 1987, 2012). A comparable assessment of students transitioned to a PBL curriculum did not show any identifiable difference on the USMLE scores ( Thompson et al, 2013). Assessment of students graduated from an organized PBL curriculum is better prepared for clinical vignette questions that required problem information analysis ( Prihatiningsih and Qomariyah, 2016). Thompson (2013) recognized there is an adjustment period of several semesters or years to accept the innovative teaching strategies and achieving widespread effectiveness.There is strong evidence that PBL pedagogy will better prepare students for physician competencies. But the same graduates identified themselves to possess less medical knowledge than graduates from the Lecture-Based Learning medical school ( Koh et al, 2008). PBL encourages critical-thinking in medical education and achieving higher cognitive integration ( Albanese and Mitchell, 1993; Nandi et al, 2000; Smits et al, 2002; Newman, 2003). Schmidt and Boshuizen (1993) found that basic science knowledge is necessary and fundamental to problem-solving in clinical settings.

5. **Structures, Characteristics and Functions of the PBL unit:**

A majority of medical schools around the world have attempted to implement PBL pedagogy either as the supplemental delivery of an individual course or as the mainstay of the entire curriculum ( Whyte, 2016). In most medical school, PBL is usually a portion of the integrated pedagogy within the medical curriculum to accomplish the learning objectives in knowledge, skills, and higher learning attributes. Majority of the medical school will supplement additional number of classroom lectures to introduce pre-requisite or difficult core-discipline materials in concurrent with the PBL curriculum ( Wood, 2003; Whyte, 2016). Majority of the PBL sessions are structured on the Maastricht “seven jump” progression, a variation of the format is also being introduced ( Schmidt, 1983; Barrett and Cashman, 2010).A typical functional PBL unit consists of a group of eight to ten students working on the case-based problem. At the formation of the group, students are assigned to the roles of chairperson, reader, scribe, and a timekeeper while a tutor facilitates the entire process over multiple sessions ( Barrett and Cashman, 2010). The same group of students and tutor are together long enough to develop a good group dynamics allowing necessary adjustments to emerging dysfunctional behaviors and personality clashes ( Barrows, 1994; Barrett and Cashman, 2010; Whyte, 2016).

The success of the PBL unit depends on the entire fundamental concepts described above. The PBL case-scenario must be of high quality, which follows the basic science discipline content learning objectives that have been identified by the course directors. The PBL case designer must possess the understanding of the Miller’s Prism, which is incorporated in the Bloom’s Taxonomy with the attainment of cognition, behavior, and skills to perform as a competent clinician ( O’Leary, 2015). Miller (1990) stated that the “knows” at the base of the pyramid assured the learned knowledge is attained to demonstrate professional competencies effectively. The PBL cases must standardize to the educational level and reflect the progressive attainment of competencies at each level of Miller’s pyramid. Each week students should have sufficient time to do self-directed learning as required according to the PBL session ( Wood, 2003).

6. **The Tutor/Facilitator Experience and Competencies:**

Student-focused PBL is resource intensive on the institution and faculties. Traditional educators are providers of information and new disciplines are required as greater demands on tutors to facilitate, encourage, and lead a small PBL unit into a proactive self-learning atmosphere ( Groves, 2005; Walsh, 2005; Leary et al, 2009). The role of the tutor/facilitator is to facilitate the PBL unit to identify important hypotheses and achieve appropriate course learning objectives effectively according to the group dynamics (Dolmans and Wolfhagen, 2005). The tutor must be familiar with the expected outcome and actively engage in step 7 of the process to enable the success of the PBL scenario and ensuring that all the students have achieved self-directed learning goals ( Wood, 2003). Tutors should have a clear outline to lead students to the required content learning objectives in order to gain the basic knowledge that is required to solve the PBL case. Faculty development is critically essential and it must focus on the institution’s educational strategy and the discipline course learning objectives within the PBL module context designed for the entire curriculum ( Swanson et al, 1990).This will empower the PBL tutors to be an effective facilitator with sound supervisory aptitude needed to resolve issues that may arise from a dysfunctional PBL unit ( Hmelo-Silver and Barrows, 2006).

Schmidt (1993) advocates content experts are better tutors. Swanson (1990) believes it is not necessary and some have mixed feelings claiming that expert with content knowledge is important, but it is secondary to other factors ( Hmelo-Silver and Barrows, 2006). It is without the doubt that tutors are vital to the success of a PBL unit. Based on the research data, an expert with content knowledge may not be critical but both subject-matter knowledge and process-facilitation skills (mixed) are necessary characteristics of an effective tutor ( Swanson et al, 1990). Leary (2009) showed that the mixed and peer tutors do better than instructor tutors and automated tutors consistently do better than all other tutors. Automated tutors are better because of its consistency, standardization and reliability to a set program.

7. **The Objective and Frequency for an Effective Student Assessments:**

The importance of assessment is to validate content teaching, learning outcomes, and professional activities to support reliability and avoid misinformed student knowledge attainment ( Vleuten et al, 2017).This requires referenced assessment that will adopt a holistic and divergent attitude involving a greater representation of peer and self-assessments that will reflect the higher self-learning in PBL ( Biggs, 2007).The significance of the PBL assessment should reflect the higher learning that is representative of the student’s future professional career and the lifelong learning skills required to develop, adapt, and prepare in a rapidly changing profession ( Macdonald and Savin-Baden, 2004).

Macdonald and Savin-Baden (2004) have offered several holistic and divergent assessments that will fit well with problem-based learning. A list of PBL assessments should focus on: (1) practice context; (2) process based professional activity; (3) student’s developmental progression; (4) professional assessment capacity; (5) engage in self-awareness and self-directed learning; (6) validity between learning objectives and anticipated outcome competency according to the adopted PBL learning and teaching methods.Same authors also offered a list demonstrating some of the successful assessments that have been used in PBL.

PBL requires a very different understanding to the assessment as learning and teaching. The success of the assessment will reflect the higher learning designed in PBL to drive the desired type of self-directed learning in students ( Macdonald and Savin-Baden, 2004). Biggs (2007) believed this assessment requires decisive factors rather than the traditional norm.It should adopt the holistic and divergent attitude that will involve a greater representation of peer and self-assessments and all features reflecting PBL pedagogy ( Macdonald and Savin-Baden, 2004).Assessments should reveal the student’s cognitive contexts showing how they would cope, act, think and strive for higher learning needed to continue and develop a lifelong medical career ( Biggs, 2007; Macdonald and Savin-Baden, 2004).

If the tutor uses the same traditional curriculum pedagogy assessments to PBL, the outcome assessment can be misconstrued between PBL objectives and the learning outcomes ( Vleuten et al, 2017). Many of the unfavorable side effects about the content-based knowledge assessments seem to undermine or contradict good intentions of effective learning. Such side effects include short-term memorization without understanding, mnemonic rather than reasoning, encourages truancy to low yield sessions and total avoidance of responsibilities on the basis of assessment preparation ( Berendonk et al, 2013). Unfortunately, the content-based knowledge examinations are prevalent among professional licensure governing entities and they are slowly adapting to the concept of PBL assessments. The responsibility of any institution is to provide a solid foundation to Barrows’ “key educational objectives” and succeeding to the chosen career path of a student, passing the licensing exam.

The designed USMLE like licensure exams are assessments involving a range of intellectual and cognitive memories in a variety of required disciplines ( Farquhar et al, 1986; Enarson and Cariaga-Lo, 2001).The solution came to Maastricht medical school in Netherland when there was a need to standardize and validate the student knowledge-domain achievements. In 1977, a progress test, “Comprehensive Basic Science Examination (CBSE)” a formative assessment was developed in a true/false and don’t know format to assess the knowledge attainment over the course of a student’s medical education ( Vleuten et al, 2009). It is designed to sample content knowledge across all basic-science disciplines relating to medical degree relevance. A newly constructed progress test is given four times annually regardless of the students’ class and knowledge level. The progress test is found to be a reliable longitudinal measure of the validity in content delivery to knowledge achievement at every educational level of the student in PBL or traditional curriculum ( Vleuten et al, 2009; Johnson et al, 2014; Tontus & Midik, 2017).

8. **Self PBL Program Assessments:**

Shamsan B (2009) performed an internal PBL program self-assessment evaluation indicated that students are bored after 18 months of the same repetitious routine of the learning process. Students affirmed that the PBL process tends to be habitual, lacking challenging and motivating engagements thus, resulting students to skip steps. Instead of discussion and sharing of information in the second session, which has compromised to a peer-peer presentation with no collaboration made to evaluate, analyze, and formulate findings to support the differential diagnosis. Students avoided their responsibilities in active discussion out of impassiveness, laziness and minimal interest as found in some similar studies ( Dolmans et al, 2001; Moust et al, 2005).

A “Self PBL Program Assessment” should include program goals and the fundamental design of the curriculum. A SWOT analysis can be adopted to assess findings pertain to the internal organization (Strengths and Weaknesses), and the external context (Opportunities and Threats). The internal organization would examine the student needs, faculty development and teaching quality, student advising, diversity inclusiveness, student success outcome, institutional support and policy. The external context must identify the social responsibility, scholarly research, discipline effectiveness, and stakeholder relationships.

The overall strategy is to increase PBL educational effectiveness by addressing its weaknesses and respond to opportunities and threats by strengthening the Standardization, Reliability and Validity of the program. I am proposing a holistic and divergent approach to “Self PBL Program Assessment”. It must include these four strategic priorities: 1) Continuous improvement of the PBL Program as it reflects to achieve PBL objectives, improve and integrate content-knowledge, student interests and interactions, tutor experience and development, formative and summative assessments; 2) Innovative learning within PBL unit where students will remain a high priority through active sharing and securing of knowledge; 3) Ongoing attention to critical priorities with a strong focus on the PBL process and continuous re-evaluation of the PBL program; 4) Re-affirm the institution and stakeholders’ continuous support and ensuring PBL goals are aligned to the core mission and vision.

### Standardization in PBL Process as Relates to the Fundamental Roots:

The nature of unguided or minimally guided curricular process ignores the orderly and standardized approaches to both; (1) the logical organization that comprises human cognitive assimilation and (2) research evidence indicate that guided learning is more effective and efficient than the unguided process. The advantage of facilitator guidance recedes when the learner has sufficiently attained greater knowledge, thus, the learner will achieve “auto-guidance” in self-learning ( Kirschner et al, 2006).The ultimate outcome of a standardized PBL program is twofold. It enables individual student benefiting from team cohesiveness and collaboration within a PBL unit, and it allows comparable achievement of student’s cognitive attainment between PBL units in a medical school ( Moust et al, 2005).

The MedBiquitous Consortium is an accredited developer of information technology using XML schema specifications to create a blueprint for continuous improvement and lifelong learning for quality healthcare in all three medical education levels of undergraduate, postgraduate and health professionals. The standards of competency, learning and assessment activities, learning objectives are collected, shared and communicated across all healthcare professional educations. Shamsan and Syed (2009) have identified and selected three technical standards of Curriculum Inventory (CI), Competency Framework (CF) and Competency Objectives (CO) to assist in the reporting and communication of standardization, reliability and validity of a medical program.

These technical standards are the basis for comparative analysis and it will help to unite (mapping) all related curriculum educational contents to learning, teaching, and assessment methods. The core-discipline course content “Learning Objectives” (identified by the curriculum committee) are the desired higher education competency goals of any medical program. Tutors should have the experience to facilitate the PBL unit process and ensuring students to maximize and achieve their intended learning objectives.

The design of PBL pedagogy in medical education emphasizes on training graduates to deliver high-quality patient-centred care and integrate into an increasingly demanding health care system ( Neufeld and Barrows, 1974).The PBL units are multiple entities of self-learning sub-units attempting to achieve the same goal in a variety of self-directed pathways guided by tutors with different backgrounds ( Barrows, 1994).The PBL units are lacking standardization within the same institution ultimately fail to achieve the same goal ( Moust et al, 2005).

The authors of this paper will interlink the relationship of the three technical standards (CI, CF and CO) to the eight fundamental roots. The standardization factor of any medical program is encompassed and intertwined between the CI and CF. The student evaluation and assessment methodology are emphasized in the reliability and validity of CO. Ongoing changes, evaluation and re-organization of the fundamental roots will ultimately affect the standardization, reliability and validity of the PBL units and their ability to achieve comparable education as one PBL Program.

By examining the PBL fundamental roots, I have identified the Curriculum Inventory (CI) as roots 4-7. The most important root is (4) “PBL Learning Objectives must include Content Learning Objectives from Required Disciplines”; the Learning Objectives must be clearly identified as the ultimate student goal within every level of the PBL case. The Competency Framework (CF) involving roots 1-3, 5 and 6. The initiation, implementation and conceptual design of the PBL process and framework in 1-3 and 5 have already been accomplished in PBL established program and they require the ongoing program re-evaluation (#8) to identify program strengths and weaknesses. The most cause-effective and ongoing CF is the standardization of the tutors (#6). Tutor experience, consistency and effectiveness in leading students to Learning Objectives are crucial to the comparable success of the PBL unit ( Leary, 2009; Walsh, 2005; Groves, 2005).Tutors must be knowledgeable and work within the facilitator guidelines supported by continuous and on-going faculty development, on-site training and supervision, and evidence-based information with the latest research knowledge in PBL ( Schmidt et al, 1993). ( Table 1)

**Table 1.** Interlinking of Fundamental Roots to Technical Standards

Fundamental roots	Description	Technical Standards
1	The Institutional acceptance to change and adjustment to PBL ownership	StandardsCF
2	The PBL objectives as it reflects on institutional mission and vision	StandardsCF
3	Student abilities and diversities	StandardsCF
4	PBL learning objectives must include content Learning objectives from required disciplines	StandardsCI
5	Structures, Characteristics and Functions of the PBL unit	StandardsCI,CF
6	The Tutor/Facilitator Experience and Competencies	StandardsCI,CF
7	The objective and frequency for an effective student assessment	Standards (CI) Reliability and Validity (CO)
8	Self PBL program assessments	Standards (CI,CF) Reliability and Validity (CO)

### Creating Reliability and Validity in Competency Objective (CO) Assessment of a PBL Program:

Unlike Learning Objectives, Competency Objectives (CO) is defined by applied competent skills and knowledge that enable a professional clinician to successfully complete their work responsibilities. CO is the higher level in Miller’s prism ( Miller, 1990).The ultimate success of an educational program is the attainment and ongoing refinement of a student’s Competency Objectives ( Koh et al, 2008). The reliability and validity of the CO are identified as the root #7 “The Objective and Frequency for Effective Student Assessments”. The assessment process must be comparably standardized and designed to be effectively delivered within each PBL unit achieving reliability and validity in the PBL educational program as a whole.

Vleuten (2017) has exquisitely summarized the changing philosophy of assessments along the evolution of education from teacher-focused to student-focused learning. The “Assessment of Learning” directives, the aim is to promote an accurate knowledge-based response from our learners using various methodologies known in the literature as the summative assessment. The importance is the credibility of student learning as applied to the decision-making function of assessment ( Segers et al, 2003). The “Assessment for Learning” is synonymous to “formative assessment”. The aim of the assessment is to evaluate the outcome on facilitated student-based learning ( Black and Wiliam, 2009). The effectiveness of the formative assessment strategies is inclusive of but not limited to self-assessment, 360 ^0^ feedback, peer-peer assessment and rubrics. In an “Assessment as Learning” directives both the cognitive-decision assessment and the student-based learning assessment are integrated into one definable combined assessment ( Clark, 2010). A well-designed Outcome assessment is the fundamental goals to reliability and validity. A complete and appropriate assessment program is aimed to achieve comparable PBL units with effective student-based learning and optimizing accurate content knowledge attainment.

Almost all PBL programs have adopted the “Assessment for Learning” that are feedback-oriented and continuous longitudinal evaluations throughout the entire semester that can vary on a “day-to-day” continuum. It is an informal assessment activity that consciously organized as a holistic and divergent assessment ( Shavelson et al, 2008). The personal experience and relationship developed in the PBL unit will influence the overall judgment of the person being assessed ( Berendonk et al, 2013).Therefore, human bias is an undeniable form of a holistic assessment. A reliable method to reduce bias is to standardize the assessment process and by identifying strict assessment criteria in a checklist enforced with a training program to standardize the assessors.Each individual assessment data is low stake that serves to provide information on the learner participation within the PBL unit engagement. These holistic and divergent evaluation assessments reflect the student’s participation in PBL unit and fail to demonstrate the true knowledge acquired specified within the PBL content Learning Objectives with no reliability and validity to the Learning Objectives reflected in the Miller’s pyramid of Cognitive Competency ( Vleuten et al, 2017).

A full PBL medical education program should be structured according to a competency framework that is outlined in the curriculum and the assessment design fitting to the medical program ( Frank, 2007, 2015). A reliable and valid assessment must accomplish the holistic and divergent approach of the PBL unit and the competency objectives of the PBL program. One form of a longitudinal content competency assessment is the use of progress testing ( Wrigley et al, 2012). One could use a progress test as a formative or summative examination, but the same progress test should be administered to all level and seniority of students within the same medical school every three months. Students cannot study or prepare for a progress test because anything can be asked. Student scores improve every semester according to their educational level.Progress testing will provide valuable feedback on student cognitive domain attainment without the shortfall of test directed studying and neglecting the responsibility of the PBL unit sessions ( Vleuten et al, 2009).

The “Assessment as Learning” directives optimize student learning and decision making assessments. The longitudinal “Holistic and Divergent approach” of self, peer and tutor assessments (i.e. direct observation, 360 ^0^ feedback, video recording, work-assignments and project assessments) will provide a wealth of information to assess the student within the competency framework of the PBL unit. Meanwhile, a longitudinal “Progress Testing”, a formative or summative assessment is implemented to assess the cognitive attainment over the entire career of the student. The reliability and validity of the longitudinal “Assessments as Learning” facilitate self-directed learning by monitoring self-reflection from a 360 ^0^ assessment feedback and a Progress Testing of the cognitive-attainment domain. Progress testing should be included as an intricate part of a complete curriculum assessment. When “Assessment as Learning” is fully integrated with the cognitive-learning strategy, then the learners will achieve and become a competent professional ( Altahawi et al, 2012).

## Conclusion

The conclusion of the illusive PBL program success is still out with the jury. Over the last half decade of reviews and research reports, they all have focused on a specific section of the PBL program. In this paper, I have identified eight fundamental roots of a PBL program. The PBL program is comprised of multiple units. The success of the PBL program must be evaluated from the standardization, reliability and validity of these fundamental roots of the program and not as an individual unit.

## Take Home Messages

• Over half of all medical schools globally have introduced various versions of PBL pedagogy in their medical education program achieving assorted modifications of outcomes.
• The importance of Standardization must be considered when incorporated into the fundamental framework of a PBL program to regulate all PBL units in a unified outcome.
• The success of any PBL program must be evaluated as a whole from the perspective of Standardization, Reliability and Validity working synchronously between fundamental roots and not the individual PBL unit.
• The reliability and validity of the Competency Objective assessment process must be comparable within each PBL unit and educational program as a whole.
• The learners will achieve and become a competent professional when “Assessment as Learning” is fully integrated with the cognitive-learning strategy.

## Notes On Contributors

Dr. Lee HangFu is the Associate Dean of Clinical Academic Affair with the Windsor University School of Medicine. His major accomplishment is the ongoing concept of the “One University” cohesiveness. He has amalgamated the school Clinical Curriculum Program (inclusive of PBL, TBL, and SBL), Policies, Learning Objectives, OSCE Advancement and Assessments, and Psychometric Analysis between Chicago, Houston, Jamaica and St. Kitts. He has collaborated with the Preceptors, Clinicians and Departmental Chairs through Faculty Development for a comparable clinical education in standardization, reliability and validity as required by the Accreditation Committee. ORCID: https://orcid.org/0000-0001-7626-6268.

Dr. Samal Nauhria M.D. Pathology is Chair of Pathophysiology and his current research is based on the use of technology in medical education.

## References

[ref1] Albanese M. A. and Mitchell S. (1993) Problem-based learning: a review of literature on its outcomes and implementation issues. Acad Med. 68(1), pp.52–81. https://www.ncbi.nlm.nih.gov/pubmed/8447896 8447896 10.1097/00001888-199301000-00012

[ref2] Altahawi F. Sisk B. Poloskey S. Hicks C. (2012) Student perspectives on assessment: experience in a competency-based portfolio system. Med Teach. 34(3), pp.221–225. 10.3109/0142159X.2012.652243 22364454

[ref3] Azer S. A. (2001) Problem-based learning. A critical review of its educational objectives and the rationale for its use. Saudi Med J. 22(4), pp.299–305. https://www.ncbi.nlm.nih.gov/pubmed/11331485 11331485

[ref4] Barrett T. and Cashman D. (eds.) (2010) A Practitioners’ guide to enquiry and problem-based learning. Dublin: UCD Teaching and Learning.

[ref5] Barrows H. and Tamblyn R. (1988) Problem-based learning: An approach to medical education. New York: Springer Publishing Company.

[ref6] Barrows H. S. (1986) A taxonomy of problem-based learning methods. Med Educ. 20(6), pp.481–486. https://www.ncbi.nlm.nih.gov/pubmed/3796328 3796328 10.1111/j.1365-2923.1986.tb01386.x

[ref7] Barrows H. S. (1994) Practice-based learning: problem-based learning applied to medical education. Southern Illinois University School of Medicine.

[ref8] Berendonk C. Stalmeijer R. E. and Schuwirth L. W. (2013) Expertise in performance assessment: assessors’ perspectives. Adv Health Sci Educ Theory Pract. 18(4), pp.559–571. 10.1007/s10459-012-9392-x 22847173 PMC3767885

[ref9] Berkson L. (1993) Problem-based learning: have the expectations been met? Acad Med. 68(10 Suppl), pp.S79–S88. 10.1097/00001888-199310000-00053 8216642

[ref10] Biggs J. B. and Tang C. S. (2007) Teaching for quality learning at university : what the student does. 3rd edn. Maidenhead: Open University Press.

[ref11] Black P. and Wiliam D. (2009) Developing the theory of formative assessment. Educational Assessment, Evaluation and Accountability(formerly: Journal of Personnel Evaluation in Education). 21(1), p.5. 10.1007/s11092-008-9068-5

[ref12] Clark I. (2010) Formative Assessment: ‘There is Nothing so Practical as a Good Theory’. Australian Journal of Education. 54(3), pp.341–352. 10.1177/000494411005400308

[ref13] Colliver J. A. (2000) Effectiveness of problem-based learning curricula: research and theory. Acad Med. 75(3), pp.259–266. https://www.ncbi.nlm.nih.gov/pubmed/10724315 10724315 10.1097/00001888-200003000-00017

[ref14] Das Carlo M. Swadi H. and Mpofu D. (2003) Medical student perceptions of factors affecting productivity of problem-based learning tutorial groups: does culture influence the outcome? Teach Learn Med. 15(1), pp.59–64. 10.1207/S15328015TLM1501_11 12632710

[ref15] Dolmans D. H. Wolfhagen I. H. van der Vleuten C. P. and Wijnen W. H. (2001) Solving problems with group work in problem-based learning: hold on to the philosophy. Med Educ. 35(9), pp.884–889. 10.1046/j.1365-2923.2001.00915.x 11555227

[ref16] Duek J. (2000) Whose Group is it anyway? Equity of Student Discourse in problem-based learning (PBL).in Hmelo C. and Evensen D. (eds.) Problem-based learning, A research perspective on learning interactions. London: Lawrence Erlbaum Associates, pp.75–107.

[ref17] Enarson C. and Cariaga-Lo L. (2001) Influence of curriculum type on student performance in the United States Medical Licensing Examination Step 1 and Step 2 exams: problem-based learning vs. lecture-based curriculum. Med Educ. 35(11), pp.1050–1055. 11703641 10.1046/j.1365-2923.2001.01058.x

[ref18] Farquhar L. J. Haf J. and Kotabe K. (1986) Effect of two preclinical curricula on NBME Part I examination performance. J Med Educ. 61(5), pp.368–373. https://www.ncbi.nlm.nih.gov/pubmed/3701811 3701811 10.1097/00001888-198605000-00003

[ref19] Frank J. R. and Danoff D. (2007) The CanMEDS initiative: implementing an outcomes-based framework of physician competencies. Med Teach. 29(7), pp.642–647. 10.1080/01421590701746983 18236250

[ref20] Frank J. Snell L. and Sherbino J. (eds.) (2015) CanMEDS 2015 Physician Competency Framework. Ottawa: Royal College of Physicians and Surgeons of Canada.

[ref21] Fullan M. (1992) Successful school improvement: the implementation perspective and beyond. Buckingham: Open University Press.

[ref22] Gill E. Tuck A. Lee D. W. Beckert L. (2004) Tutorial dynamics and participation in small groups: a student perspective in a multicultural setting. N Z Med J. 117(1205), p.U1142. https://www.ncbi.nlm.nih.gov/pubmed/15570326 15570326

[ref23] Groen G. J. Norman G. R. and Patel V. L. (1993) Reasoning and Instruction in Medical Curricula. Cognition and Instruction. 10(4), pp.335–378. 10.1207/s1532690xci1004_2

[ref24] Groves M. Rego P. and O’Rourke P. (2005) Tutoring in problem-based learning medical curricula: the influence of tutor background and style on effectiveness. BMC Med Educ. 5(1), p.20. 10.1186/1472-6920-5-20 15938758 PMC1180438

[ref25] Hartling L. Spooner C. Tjosvold L. and Oswald A. (2010) Problem-based learning in pre-clinical medical education: 22 years of outcome research. Med Teach. 32(1), pp.28–35. 10.3109/01421590903200789 20095771

[ref26] Hendry G. D. Ryan G. and Harris J. (2003) Group problems in problem-based learning. Med Teach. 25(6), pp.609–616. 10.1080/0142159031000137427 15369908

[ref27] Hmelo C. E. Gotterer G. S. and Bransford J. D. (1997) A theory-driven approach to assessing the cognitive effects of PBL. Instructional Science. 25(6), pp.387–408. 10.1023/a:1003013126262

[ref28] Hmelo-Silver C. E. and Barrows H. S. (2006) Goals and Strategies of a Problem-based Learning Facilitator. Interdisciplinary Journal of Problem-Based Learning. 1(1). 10.7771/1541-5015.1004

[ref29] Husain A. (2011) Problem-based Learning: A Current Model of Education. Oman Med J. 26(4), p.295. 10.5001/omj.2011.74 22043442 PMC3191708

[ref30] Johnson T. R. Khalil M. K. Peppler R. D. Davey D. D. (2014) Use of the NBME Comprehensive Basic Science Examination as a progress test in the preclerkship curriculum of a new medical school. Adv Physiol Educ. 38(4), pp.315–320. 10.1152/advan.00047.2014 25434014

[ref31] Kaufman D. M. and Mann K. V. (1997) Basic sciences in problem-based learning and conventional curricula: students’ attitudes. Med Educ. 31(3), pp.177–180. 10.1111/j.1365-2923.1997.tb02562.x 9231134

[ref32] Kirschner P. A. Sweller J. and Clark R. E. (2006) Why Minimal Guidance During Instruction Does Not Work: An Analysis of the Failure of Constructivist, Discovery, Problem-Based, Experiential, and Inquiry-Based Teaching. Educational Psychologist. 41(2), pp.75–86. 10.1207/s15326985ep4102_1

[ref33] Koh G. C. Khoo H. E. Wong M. L. and Koh D. (2008) The effects of problem-based learning during medical school on physician competency: a systematic review. CMAJ. 178(1), pp.34–41. 10.1503/cmaj.070565 18166729 PMC2151117

[ref34] Leary H. Walker A. Fitt M. and Shelton B. (2009) Expert Versus Novice Tutors: Impacts on Student Outcomes in Problem-Based Learning. American Educational Research Association annual conference. San Diego California.

[ref35] Lee R. and Kwan C. (1997) The Use of Problem-Based Learning in Medical Education. J Med Education. 1(2), pp.11–20.

[ref36] Macdonald R. and Savin-Baden M. (2004) A Briefing on Assessment in Problem-based Learning. Heslington York: Learning and Teaching Support Network (LTSN).

[ref37] McLean M. Van Wyk J. M. Peters-Futre E. M. and Higgins-Opitz S. B. (2006) The small group in problem-based learning: more than a cognitive ‘learning’ experience for first-year medical students in a diverse population. Med Teach. 28(4), pp.e94–103. 10.1080/01421590600726987 16807164

[ref38] Meacham J. and Gaff J. (2006) Learning Goals in Mission Statements: Implications for Educational Leadership. Liberal Education.p.8. Available at: https://eric.ed.gov/?id=EJ743272

[ref39] Miller G. E. (1990) The assessment of clinical skills/competence/performance. Acad Med. 65(9 Suppl), pp.S63–S67. https://www.ncbi.nlm.nih.gov/pubmed/2400509 2400509 10.1097/00001888-199009000-00045

[ref40] Moust J. Roebertsen H. Savelberg H. and De Rijk A. (2005) Revitalising PBL groups: evaluating PBL with study teams. Educ Health (Abingdon). 18(1), pp.62–73. 15804646 10.1080/13576280500042705

[ref41] Nandi P. L. Chan J. N. Chan C. P. Chan P. (2000) Undergraduate medical education: comparison of problem-based learning and conventional teaching. Hong Kong Med J. 6(3), pp.301–306. https://www.ncbi.nlm.nih.gov/pubmed/11025850 11025850

[ref42] Neufeld V. R. and Barrows H. S. (1974) The “McMaster Philosophy”: an approach to medical education. J Med Educ. 49(11), pp.1040–1050. https://www.ncbi.nlm.nih.gov/pubmed/4444006 4444006

[ref43] Newman M. (2003) A Pilot Systematic Review and Meta-Analysis on the Effectiveness of Problem Based Learning.p.77. Available at: https://eric.ed.gov/?id=ED476146

[ref44] Norman G. R. and Schmidt H. G. (2000) Effectiveness of problem-based learning curricula: theory, practice and paper darts. Med Educ. 34(9), pp.721–728. 10.1046/j.1365-2923.2000.00749.x 10972750

[ref45] O’Leary F. (2015) Simulation as a high stakes assessment tool in emergency medicine. Emerg Med Australas. 27(2), pp.173–175. 10.1111/1742-6723.12370 25690440 PMC4415593

[ref46] Prihatiningsih T. and Qomariyah N. (2016) Evaluation of a Problem Based Learning Curriculum Using Content Analysis. International Journal of Evaluation and Research in Education.pp.205–10. Available at: https://eric.ed.gov/?id=EJ1115387

[ref47] Schmidt H. G. (1983) Problem-based learning: rationale and description. Medical Education. 17(1), pp.11–16. 10.1111/j.1365-2923.1983.tb01086.x 6823214

[ref48] Schmidt H. G. and Boshuizen H. P. A. (1993) On acquiring expertise in medicine. Educational Psychology Review. 5(3), pp.205–221. 10.1007/bf01323044

[ref49] Schmidt H. G. Dauphinee W. D. and Patel V. L. (1987) Comparing the effects of problem-based and conventional curricula in an international sample. J Med Educ. 62(4), pp.305–315. 3560175 10.1097/00001888-198704000-00002

[ref50] Schmidt H. G. Muijtjens A. M. Van der Vleuten C. P. and Norman G. R. (2012) Differential student attrition and differential exposure mask effects of problem-based learning in curriculum comparison studies. Acad Med. 87(4), pp.463–475. 10.1097/ACM.0b013e318249591a 22361797

[ref51] Schmidt H. G. van der Arend A. Moust J. H. Kokx I. (1993) Influence of tutors’ subject-matter expertise on student effort and achievement in problem-based learning. Acad Med. 68(10), pp.784–791. 10.1097/00001888-199310000-00018 8397613

[ref52] Segers M. Dochy F. and Cascallar E. (2003) Optimising New Modes of Assessment.In Search of Qualities and Standards. 1 edn. Springer Netherlands. 10.1007/0-306-48125-1

[ref53] Shamsan B. and Syed A. T. (2009) Evaluation of problem based learning course at college of medicine, qassim university, saudi arabia. Int J Health Sci (Qassim). 3(2), pp.249–258. https://www.ncbi.nlm.nih.gov/pubmed/21475544 21475544 PMC3068813

[ref54] Shavelson R. J. Young D. B. Ayala C. C. Brandon P. R. (2008) On the Impact of Curriculum-Embedded Formative Assessment on Learning: A Collaboration between Curriculum and Assessment Developers. Applied Measurement in Education. 21(4), pp.295–314. 10.1080/08957340802347647

[ref55] Singaram V. S. Dolmans D. H. Lachman N. and van der Vleuten C. P. (2008) Perceptions of problem-based learning (PBL) group effectiveness in a socially-culturally diverse medical student population. Educ Health (Abingdon). 21(2), p.116. https://www.ncbi.nlm.nih.gov/pubmed/19039743 19039743

[ref56] Smits P. B. Verbeek J. H. and de Buisonje C. D. (2002) Problem based learning in continuing medical education: a review of controlled evaluation studies. BMJ. 324(7330), pp.153–156. 10.1136/bmj.324.7330.153 11799034 PMC64518

[ref57] Swanson D. Stalenhoef-Halling B. and Vleuten C. v. d. (1990) Effect of tutor characteristics on test performance of students in a problem-based curriculum.in Bender W. Hiemstra R. Scherpbier A. and Zwierstra R. (eds.) Teaching and assessing clinical competence. Groningen, The Netherlands: Boek Werk Publications, pp.129–134.

[ref58] Thompson A. R. Braun M. W. and O’Loughlin V. D. (2013) A comparison of student performance on discipline-specific versus integrated exams in a medical school course. Adv Physiol Educ. 37(4), pp.370–376. 10.1152/advan.00015.2013 24292915

[ref59] Tontus H. O. and Ozlem M. (2017) Evaluation of Curriculum by Progress Test. Journal of US-China Medical Science. 14(6). 10.17265/1548-6648/2017.06.003

[ref60] Tsou K. I. Cho S. L. Lin C. S. Sy L. B. (2009) Short-term outcomes of a near-full PBL curriculum in a new Taiwan medical school. Kaohsiung J Med Sci. 25(5), pp.282–293. 10.1016/S1607-551X(09)70075-0 19502151 PMC11917576

[ref61] van der Vleuten C. Sluijsmans D. and Joosten-ten Brinke D. (2017) Competence Assessment as Learner Support in Education.in Mulder M. (ed.) Competence-based Vocational and Professional Education. Switzerland: Springer International Publishing, pp.607–630. Chapter 28. 10.1007/978-3-319-41713-4_28

[ref62] Vernon D. T. and Blake R. L. (1993) Does problem-based learning work? A meta-analysis of evaluative research. Acad Med. 68(7), pp.550–563. 10.1097/00001888-199307000-00015 8323649

[ref63] Vleuten C. P. M. V. D. Verwijnen G. M. and Wijnen W. H. F. W. (2009) Fifteen years of experience with progress testing in a problem-based learning curriculum. Medical Teacher. 18(2), pp.103–109. 10.3109/01421599609034142

[ref64] Walsh A. (2005) The tutor in problem based learning: a novice’s guide.Edited by Sciarra A. Hamilton, ON: Program for Faculty Development. McMaster University, Faculty of Health Sciences.

[ref65] Whyte R. (2016) Curriculum inventory in context. AAMC.org. [Online]. Available at: https://www.aamc.org/download/464752/data/ciic03-4apr2016.pdf( Accessed: 9 February 2019)

[ref66] Wood D. F. (2003) Problem based learning. BMJ. 326(7384), pp.328–330. https://www.ncbi.nlm.nih.gov/pubmed/12574050 12574050 10.1136/bmj.326.7384.328 PMC1125189

[ref67] Woods D. (1994) Problem Based Learning-how to gain the Most from PBL. Waterdown: W L Griffen Printing.

[ref68] Wrigley W. van der Vleuten C. P. Freeman A. and Muijtjens A. (2012) A systemic framework for the progress test: strengths, constraints and issues: AMEE Guide No. 71. Med Teach. 34(9), pp.683–697. 10.3109/0142159X.2012.704437 22905655

