# Supplementary material for: The Need for Standardization, Reliability and Validity in Fundamental Roots for a Successful Problem-Based Learning Program
Source: MedEdPublish (2016). 2019 Apr 22;8:93. [Version 1] doi: 10.15694/mep.2019.000093.1 (PMC10712582; doi:10.15694/mep.2019.000093.1)
Supplement: Peer review history

# Reviewer report: Reviewer response for version 1

**DOI:** 10.21956/mep.19831.r29887

**Published:** 2019-05-06

**Authors:** Michelle McLean

**Affiliations:** 1 Bond University

This review has been migrated. The reviewer awarded 3 stars out of 5 Thanks for this article. Like many of us, the authors have attempted to provide some clarity around what PBL really is by offering some suggestions in terms of everyone being on the same page. Like another author, some tidying up is required in terms of language. The use of the term 'PBL unit' is confusing for me as it more along the lines of programme, course, module than group or team. I would suggest using group or team as teamwork is one of the outcomes of PBL. Having worked in the area of PBL in undergraduate medical education for about 20 years, I have come to the conclusion that PBL is too sophisticated an approach at the outset of the medical programme. Other approaches such as team-based learning are probably more appropriate for young learners. PBL requires student maturity and very well trained and motivated facilitators.

Reviewer Expertise:

NA

No decision status is available

# Reviewer report: Reviewer response for version 1

**DOI:** 10.21956/mep.19831.r29885

**Published:** 2019-04-22

**Authors:** P Ravi Shankar

**Affiliations:** 1 American International Medical University

This review has been migrated. The reviewer awarded 4 stars out of 5 I read with great interest the article on problem-based learning (PBL). PBL has been widely studied and written about in the literature. The authors provide an overview of PBL primarily through the lens of studies from developed nations. Over the decades PBL has been used in a variety of settings including the developing world either as a dominant part of the curriculum or as a learning method to supplement traditional lecture-based teaching. PBL has been used in a variety of disciplines ranging from the health sciences to engineering and management. PBL fits well into an integrated basic science curriculum and could also be used to revisit the basic sciences during the clinical years. PBL serves as a good introduction to the problem solving skills and the integration of knowledge which students will use to diagnose and manage patient problems. Some countries and schools still follow a more traditional system where the ‘normal’ is learned first followed by the ‘abnormal’ which can create challenges for PBL. The diversity of the student body and cultural issues has been mentioned by the authors and has been studied in the literature. Under heading 6 ‘the tutor/facilitator experience ’I did not understand what the authors mean by an automated tutor? The various issues related to assessing students have been well addressed in the paper. PBL is now being used in medical schools in various developing nations and also in the Caribbean and the authors can mention studies from these regions also. There are a few language and spelling issues which may need correction. Though there are two authors, the word ‘I’ is often used in the manuscript. I am not sure whether the authors are describing standardizing PBL within an institution or across institutions or both. The review of literature is comprehensive though, as mentioned more studies from developing nations can be included. The article will be of great interest to all medical educators.

Reviewer Expertise:

NA

No decision status is available

# Reviewer report: Reviewer response for version 1

**DOI:** 10.21956/mep.19831.r29886

**Published:** 2019-04-22

**Authors:** Felix Silwimba

**Affiliations:** 1 University of Lusaka

This review has been migrated. The reviewer awarded 5 stars out of 5 PBL standardization, reliability and validity. I enjoyed reading through the article. it is well argued, and I recommend it to medical educators. I agree PBL contributes to making medical practitioners’ continuous learners since they must keep solving problems throughout their careers

Reviewer Expertise:

NA

No decision status is available
